# Avanafil use in patients with erectile dysfunction and co-morbidities: Clinical insights from multiple aetiologies - A case series

**DOI:** 10.51866/cr.764

**Published:** 2025-04-09

**Authors:** Shaiful Bahari Ismail, Wan Mohd Nazlee Wan Zainon, Mohd Albaihaqi Ahmad Lotopi, Amir Nazri

**Affiliations:** 1 MBBS, MMED (F Medicine), Department of Family Medicine, School of Medical Sciences, Universiti Sains Malaysia, Kubang Kerian, Kelantan, Malaysia. E-mail: shaifulb@usm.my, shaifulbahari67@gmail.com; 2 MD, MMED (F Medicine), Family Medicine Clinic, Hospital Universiti Sains Malaysia, Kubang Kerian, Kelantan, Malaysia.; 3 MBBCh, Family Medicine Clinic, Hospital Universiti Sains Malaysia, Kubang Kerian, Kelantan, Malaysia.; 4 MBBS, Family Medicine Clinic, Hospital Universiti Sains Malaysia, Kubang Kerian, Kelantan, Malaysia.

**Keywords:** Avanafil, Erectile dysfunction, Comorbidity, Quality of life, Asian people

## Abstract

Erectile dysfunction (ED) is a prevalent medical problem affecting men globally, particularly those with co-morbidities such as cardiovascular disease, hypertension, dyslipidaemia and diabetes. This case series aimed to evaluate the effects of avanafil as a treatment option for patients with ED and other co-morbidities. Eight male patients from a family medicine clinic received either 100 mg or 200 mg avanafil based on their clinical indication. Erectile function was assessed using the International Index of Erectile Function-5 (IIEF-5) and Erection Hardness Score. The outcomes included changes in the IIEF-5 score upon avanafil treatment. Our findings demonstrated a significant increase in the 200 mg avanafil treatment group, with the IIEF-5 score increasing from 14.83±9.4 to 24.17±2.6 (P<0.05). However, no significant difference was observed in the 100 mg avanafil treatment group (P=0.7903) and the overall avanafil treatment group (100 mg + 200 mg avanafil; P=0.0501). No adverse events were reported among the patients. Avanafil is considered a potentially safe and effective treatment option for ED, particularly in individuals with co-morbidities.

## Introduction

Erectile dysfunction (ED) is defined as the inability to attain or sustain an erection for satisfactory sexual intercourse.^1^ It is a common medical problem affecting men worldwide, with the global prevalence ranging from 3% to 76.5%.^2^ Its prevalence is also expected to rise by 2025, whereby 322 million men will be affected.^3^ In an analysis of the Malaysian National Health and Morbidity Survey 2019 data, the prevalence of moderate-to-severe ED was reported to be 31.6% among sexually active men aged 18 years and above.^3^

ED imposes a substantial burden on the quality of life and reduces work productivity, leading to a significant economic burden.^4^ It can be of a psychogenic nature, which is associated with various psychosocial problems such as depression, poor self-esteem, lack of confidence and limited intimacy.^4^ However, a majority of ED cases are organic, attributing to vascular, neurogenic or hormonal causes.^5^ ED is prevalent among men with poor lifestyles including smoking and underlying co-morbidities such as cardiovascular disease, hypertension, dyslipidaemia and diabetes.^6^ Previous epidemiological research has demonstrated that the prevalence of ED among patients with hypertension, hyperlipidaemia and diabetes or heart disease is 80.4%, 78.9% and 89.2%, respectively.^7^

In the management of ED, a spectrum of medical and interventional therapies exists, encompassing phosphodiesterase type 5 inhibitors (PDE5is), vacuum erection devices, penile self-injection regimens involving vasoactive drugs and penile prostheses.^8,9^ Among these options, PDE5is are preferred as the first-line therapy due to their ease of use, better efficacy and favourable adverse effect profiles.^8,9^ Among PDE5is, avanafil has been demonstrated in various preclinical and clinical studies to be superior to sildenafil in improving the International Index of Erectile Function-erectile function domain score and having a faster onset of action with a shorter time to peak response.^8,10^ Additionally, phase III studies and long-term data have indicated that avanafil is well-tolerated with low rates of adverse effects due to its higher specificity for PDE5 compared to other phosphodiesterase subtypes.^11-13^ However, there remains a lack of evidence on avanafil for the treatment of patients with ED, particularly those with co-morbidities. Hence, this case series focused on patients with ED and different co-morbidities and evaluated the effects of avanafil as a treatment option to improve their quality of life.

## Case presentation

### Patients and methods

This case series presents multiple case reports of ED with different aetiologies (organic or psychogenic). Patients with ED were examined through routine care at a family medicine clinic, and eight cases were reviewed retrospectively based on the clinical indications of avanafil. Eight male patients who consulted at the family medicine clinic were given avanafil once they were clinically indicated. Investigations were conducted based on the clinical judgement of the attending doctor, in line with standard clinical practice.

The mean age and mean body mass index of the eight patients were 55.8±9.1 years and 27.9±4.1 kg/m^2^, respectively. These patients presented with different co-morbidities and were prescribed either 100 mg or 200 mg avanafil as assessed by doctors in the clinic. The demographics, disease background and initial examination findings of the patients were assessed (Table 1). Erectile function was evaluated using the validated erectile function domain of the International Index of Erectile Function-5 (IIEF-5) and Erection Hardness Score (EHS). All consultation and treatment procedures performed were in accordance with the standard protocols of the clinic. The patients received no additional follow-up or attention beyond what is typically provided during routine clinical care, ensuring that the data collected reflected routine practice outcomes.

**Table 1:** Demographics, disease background and initial examination findings of the patients included in the case series. BMI, Body mass index; CVA, Cerebrovascular accident; ED, Erectile dysfunction; EHS, Erection hardness score; IIEF-5, International Index of Erectile Function-5; L, Left; OD, Once daily; ON, Once at night; PRN, As needed; R, Right.

Aspects	Patient 1	Patient 2	Patient 3	Patient 4	Patient 5	Patient 6	Patient 7	Patient 8
*Demographics*								
Age (year)	54	61	62	57	60	67	40	45
BMI (kg/m^2^)	23.7	29.4	20.4	26.1	30.1	30.5	30.7	32
*Disease background*								
Chief complaints	Premature ejaculation and occasional erectile dysfunction	Unsatisfactory erection for sexual intercourse	Erectile dysfunction	Inability to sustain erection for 1 year	Erectile dysfunction for the past 3 years	Erectile dysfunction for the past 1 year	Unsatisfactory erection	Unsatisfactory erection
Comorbidities	Dyslipidaemia	Dyslipidaemia Diabetes Hypertension	Diabetes	History of CVA with R residual weakness	Diabetes Hypertension Hyperlipidaemia	Hypertension Hyperlipidaemia Diabetes mellitus	Fatty liver disease Allergic rhinitis	Hypertension Dyslipidaemia Bronchial asthma
Current medications	Atorvastatin 20 mg ON Allopurinol 300 mg OD	Amlodipine/valsartan 5 mg/80 mg OD Gliclazide modified release 20 mg OD Metformin 1000 mg BD Simvastatin 20 mg ON	Metformin 750 mg OD Subcutaneous insulin/isophane insulin 18 U BD Perindopril 8 mg OD Atorvastatin 20 mg PRN for hyperlipidaemia	Cardiprin 100 mg OD Atorvastatin 40 mg ON Celecoxib 200 mg BD Paracetamol 1 g BD Neurobion 1 tab OD	Atorvastatin 20 mg ON Irbesartan 300 mg OD Metformin XR 1500 mg OD Glyxambi 25/5 mg OD	Amlodipine 10 mg OD Frusemide 40 mg OD Vildagliptin 50 mg BD Metformin XR 2000 mg OD Gliclazide MR 120 mg OD Simvastatin 20 mg ON	Loratadine 10 mg OD	Amlodipine 10 mg OD Atorvastatin 20 mg ON Irbesartan 300 mg OD
ED duration	1 year	1 year	3 years	1 year	3 years	1 year	2 years	1 year
Tobacco use	Non-smoker	Non-smoker	Ex-smoker	Non-smoker	Ex-smoker	Non-smoker	Non-smoker	Non-smoker
*Examination findings*								
IIEF-5 score	22	18	5	18	20	8	15	17
EHS	2-3	2-3	2	2-3	2-3	2-3	3	3
Testicular size	15 cc (L) 20 cc (R)	20 cc (L) 20 cc (R)	25 cc (L) 25 cc (R)	25 cc (L) 25 cc (R)	25 cc (L) 25 cc (R)	25 cc (L) 20 cc (R)	12 cc (L) 15 cc (R)	25 cc (L) 20 cc (R)

### Ethics disclosure

This case series was exempted from ethical review by the University’s Research Ethics Committee.

### Statistical analysis

All data obtained were analysed using GraphPad Prism version 9.0 (GraphPad Software Inc., USA). All data were expressed as means ± standard deviations (SDs). One-way analysis of variance was performed to compare different experimental groups. The data were considered significant when the P-value was less than 0.05.

## Results

### Case 1

Patient 1 was a 54-year-old technician who recently remarried after his first wife passed away. He presented with complaints of premature ejaculation and occasional ED lasting less than 5 min. With a history of gouty arthritis and dyslipidaemia, the patient was taking allopurinol 300 mg OD and atorvastatin 20 mg ON, respectively. He was a non-smoker, non-vaper, non-alcoholic and non-drug user. Initial assessments revealed an IIEF-5 score of 22 and an EHS of 2-3, with normal penile examination findings. Considering his condition and marriage status, the patient was diagnosed with psychogenic ED. The management strategy involved lifestyle modifications including a low-cholesterol diet, alongside avanafil 100 mg PRN. After 1 month of intervention, there was a notable improvement with an onset time of 15 min, resulting in an enhanced IIEF-5 score of 25 and an EHS of 3-4. The patient reported satisfaction with his erections and continued the use of avanafil 100 mg PRN.

### Case 2

Patient 2 was a 61-year-old pensioner with a history of controlled hypertension, diabetes mellitus and hyperlipidaemia, presenting with a chief complaint of an inability to sustain a satisfactory erection during sexual intercourse for the past year despite being able to achieve erections. He was taking amlodipine/valsartan 5 mg/80 mg OD for hypertension, gliclazide modified release 30 mg OD and metformin 1000 mg BD for diabetes and simvastatin 20 mg ON for hyperlipidaemia. Initial assessments revealed an IIEF-5 score of 18 and an EHS of 2-3, indicative of vascular-related ED. Previous treatment with sildenafil 100 mg PRN for 3 months yielded no improvement. With a history of seven children and sexual intercourse occurring once per week, the patient’s management included lifestyle modifications such as encouraging physical activity, alongside avanafil 100 mg OD. After 1 month of follow-up, the intervention resulted in an onset time of 15 min, an improved IIEF-5 score from 18 to 23 and an EHS of 3. Despite the initial improvement, the patient expressed dissatisfaction with erection hardness. Consequently, the dosage of avanafil was increased to 200 mg PRN. After another month, we noted further improvement in his IIEF-5 score to 26 and sustained satisfaction without reported side effects.

### Case 3

Patient 3 was a 62-year-old pensioner with a history of type 2 diabetes mellitus for the past decade, presenting with a chief complaint of ED persisting for 3 years, coinciding with the time he remarried following the passing of his first wife. Despite normal libido, he noted difficulty achieving full erections for penetration. Initial treatments with sildenafil 50 mg PRN and tadalafil 20 mg PRN showed no response. Additionally, he had been living with diabetes for the past decade and was a chronic smoker but was visiting a smoking cessation clinic for the past 2 months. The patient was on metformin 750 mg OD and subcutaneous insulin/isophane insulin 18 U BD for diabetes control, perindopril 8 mg OD for hypertension and atorvastatin 20 mg ON for hyperlipidaemia. Upon examination, the patient had an initial IIEF-5 score of 5 and an EHS of 2. His management included lifestyle modifications such as exercise and avanafil 200 mg PRN. Follow-up at 1-month postintervention revealed a duration of sustenance of 2-3 min, an improved IIEF-5 score of 21 and an EHS of 3. The patient reported satisfaction with the treatment outcome and the ability to engage in sexual intercourse. No adverse effects were noted.

### Case 4

Patient 4 was a 57-year-old pensioner who was married to two spouses. He presented with a complaint of ED persisting for 1 year. Despite occasional attainment of penile hardness, it was unsustainable for more than 3-4 min. The patient reported experiencing headaches after the administration of 100 mg sildenafil. His medical history revealed a cerebrovascular accident with residual right-sided weakness, prolapsed intervertebral disc, right frozen shoulder and hyperlipidaemia. Initial evaluation showed an IIEF-5 score of 18 and an EHS of 2-3. Management encompassed lifestyle modifications including Kegel exercises and avanafil 100 mg PRN. Follow-up assessment after 1 month demonstrated an improved IIEF-5 score of 23 and an EHS of 3. Although the patient also experienced headaches with avanafil, they were reported to be milder and more tolerable than those induced by sildenafil.

### Case 5

Patient 5, a 60-year-old retired police officer, presented with a complaint of ED persisting for 3 years. Despite an initial EHS of 3, he was unable to sustain an erection for more than 3 min, leading to dissatisfaction from his spouse. Previous attempts with ED supplements purchased from social media proved to be ineffective. Initiation of sildenafil 50 mg PRN 3 months prior resulted in an improved EHS of 3-4, lasting more than 5 min, with subsequent satisfaction reported by his partner. His medical history included hypertension, hyperlipidaemia and diabetes, managed with medication. Initial evaluations revealed an IIEF-5 score of 20 without treatment and 23 on sildenafil 50 mg PRN and an EHS ranging from 2 to 3. Management included lifestyle modifications such as Kegel exercises and weight reduction, alongside avanafil 100 mg PRN. However, the patient reported no improvement with avanafil 100 mg PRN during the second visit, prompting an increase to avanafil 200 mg PRN. Subsequent assessments showed satisfaction with avanafil 200 mg PRN, with an EHS of 3-4 and an IIEF-5 score of 24, leading to the patient’s request to continue this regimen.

### Case 6

Patient 6, a 67-year-old retired police officer, presented with a chief complaint of ED persisting for 1 year, characterised by an EHS of 2 and difficulty penetrating during sexual intercourse, leading to dissatisfaction from his wife. Occasionally, he reported an EHS of 3 but lacked sustainability and confidence during sexual intercourse. He resorted to purchasing medication over the counter, including sildenafil 100 mg once a week, claiming temporary improvement. His medical history included hypertension, hyperlipidaemia and diabetes, managed with multiple medications. Initial assessments revealed an IIEF-5 score of 8 and an EHS of 2-3, indicating ED secondary to vascular causes. The IIEF-5 score did not improve with 100 mg avanafil, as reported during the 1-month follow-up. Therefore, 200 mg avanafil was administered, resulting in an improvement in the IIEF-5 score to 24 at the subsequent follow-up.

### Case 7

Patient 7, a 40-year-old state officer, presented with a chief complaint of unsatisfactory erection persisting for 2 years, characterised by gradual onset and intermittent difficulties in performance during vaginal penetration, as reported by his wife. The patient maintained good libido with morning erections occurring occasionally. His medical history included fatty liver managed with lifestyle modifications and allergic rhinitis managed with loratadine 10 mg OD, with no family history of note. Initial assessments revealed an IIEF-5 score of 15 and an EHS of 3. His young age, the intermittent occurrence of ED, the presence of morning erections, good libido and the absence of chronic comorbidities were suggestive of psychogenic ED. Management involved lifestyle modifications, including weight reduction, alongside avanafil 100 mg PRN. Despite the patient’s perception of no improvement with avanafil 100 mg PRN, his IIEF-5 score improved from 25 to 28, which is the maximum IIEF-5 score indicating normal erectile function. Consequently, due to the patient’s perception of no improvement, the dosage was increased to avanafil 200 mg PRN. Following this, the IIEF-5 score was maintained at 28, with the patient reporting improved satisfaction and no adverse events observed. The factors contributing to the patient’s condition included work-related stress, health concerns such as fatty liver, unsatisfactory erection, mild performance anxiety and a lack of confidence.

### Case 8

Patient 8, a 45-year-old teacher, presented with a chief complaint of unsatisfactory erection persisting for 1 year with a gradual onset. Although still able to achieve erections for vaginal penetration and experiencing morning erections, the patient reported dissatisfaction. Medical history included hypertension, hyperlipidaemia and bronchial asthma, managed with medications including amlodipine 10 mg OD, atorvastatin 20 mg ON and irbesartan 300 mg OD. Initial assessments revealed an IIEF-5 score of 17 and an EHS of 3, indicating ED likely of psychological origin with a vascular cause as a differential consideration. Management involved lifestyle modifications such as increased exercise and weight reduction, alongside avanafil 200 mg PRN considering his relatively young age. During the second follow-up, the patient expressed satisfaction, with an onset time of 15 min, an EHS of 3-4 and an improved IIEF-5 score from 17 to 22. At the third follow-up, he was satisfied with his erections, with an IIEF-5 score of 23, and his confidence increased.

### Effects of avanafil as a treatment option for ED

**Figure 1:**
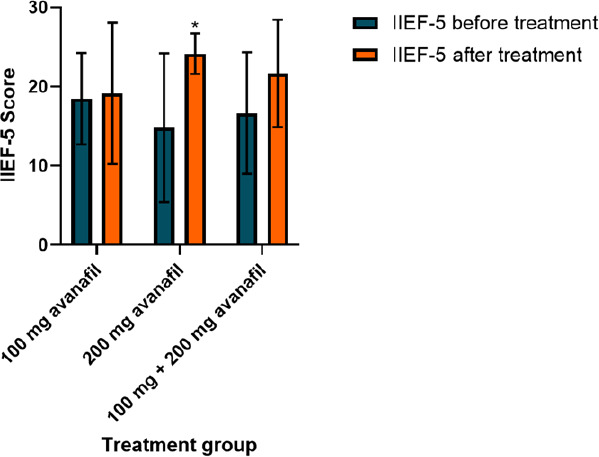
Effects of avanafil treatment on the IIEF-5 score among the patients with ED and comorbidities. Each set of bar graphs represents the IIEF-5 score before and after 100 mg avanafil treatment, 200 mg avanafil treatment and 100 mg + 200 mg avanafil treatment. *P<0.05 compared to the IIEF-5 score before treatment. ED, Erectile dysfunction; IIEF-5, International Index of Erectile Function-5

The effects of avanafil for treating ED was determined by assessing the IIEF-5 score before and after treatment. There was a significant increase in the 200 mg avanafil treatment group, whereby the IIEF-5 score increased from 14.83±9.4 to 24.17±2.6 (P<0.05; Figure 1 and Table 2). However, there was no significant difference in the 100 mg avanafil treatment group (P=0.7903), with IIEF-5 scores of 18.50±5.8 and 19.17±8.9 before and after avanafil treatment, respectively. Furthermore, the overall 100 mg + 200 mg avanafil treatment group exhibited a slight increase in the IIEF-5 score from 16.67±7.7 to 21.67±6.8 (P=0.0501). Taken together, these results suggest that 200 mg avanafil is effective in treating patients with ED and co-morbidities.

**Table 2:** Effects of avanafil treatment on the IIEF-5 score of the patients with ED and comorbidities. ED, Erectile dysfunction; IIEF-5, International Index of Erectile Function-5; SD, Standard deviation

Patient number	IIEF-5 score before treatment	IIEF-5 score after treatment
*100 mg avanafil*		
Patient 1	22	25
Patient 2	18	23
Patient 4	18	23
Patient 5	20	10
Patient 6	8	6
Patient 7	25	28
Means SD	18.50±5.8	19.17±8.9
*200 mg avanafil*		
Patient 2	23	26
Patient 3	5	21
Patient 5	10	24
Patient 6	6	24
Patient 7	28	28
Patient 8	17	22
Means SD	14.83±9.4	24.17±2.6
*100 mg + 200 mg avanafil*		
Mean ± SD	16.67±7.7	21.67±6.8

## Discussion

ED is a prevalent health condition affecting men globally, impacting their quality of life, work productivity and overall well-being.^4^ Avanafil, a second-generation PDE5i, has gained attention for its rapid onset of action and efficacy in real-world settings.^10,12-14^ It prevents detumescence by competitively binding to the catalytic site of PDE5, thereby inhibiting the degradation of cGMP Elevated cGMP levels induce relaxation of vascular smooth muscles in the corpus cavernosum, leading to dilation of penile arterial vessels and therefore resulting in an erection.^9-13^ This case series highlights the effectiveness of 200 mg avanafil in treating ED, reflected by the significantly increased IIEF-5 scores, particularly in patients with co-morbidities.

In this case series, patients with ED and co-morbidities such as gouty arthritis, dyslipidaemia, hypertension and diabetes mellitus were included. Avanafil was administered once daily at two dosages: 100 mg and 200 mg. There were limited data on 50 mg avanafil; hence, they were not included in this study.^12,15^ In contrast to other reports, the 100 mg dosage did not yield significant improvements in the IIEF-5 scores in this case series.^12^ Furthermore, two patients (Patient 05 and Patient 06) exhibited reduced IIEF-5 scores following the 100 mg avanafil regimen. However, the 200 mg avanafil dosage consistently led to significant improvements in the IIEF-5 scores across all treated patients. Notably, the patients who initially exhibited reduced IIEF-5 scores on the 100 mg regimen (Patient 05 and Patient 06) experienced better outcomes when the dosage was escalated to 200 mg. The 200 mg dosage also demonstrated a significant improvement in Patient 03 with severe ED. However, no significant difference was observed between the 100 mg and 200 mg dosages, similar to previous reports.^12,14^

The therapeutic efficacy of avanafil was observed across a diverse patient population in this case series, including those with co-morbidities such as gouty arthritis, dyslipidaemia, hypertension, diabetes mellitus and hyperlipidaemia. Furthermore, no adverse events or sudden death occurred after avanafil administration, with no noteworthy changes in blood pressure or heart rate, which is consistent with findings from the literature.^16^ This suggests that avanafil could be a viable and safe therapeutic option for ED in patients with these conditions.

While avanafil was generally well-tolerated, some patients reported mild side effects such as headaches. Indeed, headache and flushing are the most common adverse effects reported in several clinical trials.^12,17-19^ The occurrence of these side effects after avanafil administration can be attributed to cross-reactivity with PDE1.^8^ However, these side effects were deemed more tolerable than those induced by other PDE5is, due to the increased selectivity for PDE5 of avanafil compared to other PDE5is such as sildenafil and vardenafil.^9,13,17^ This suggests that avanafil may be a more suitable choice for patients who are sensitive to the side effects of PDE5is. Additionally, previous reports have suggested that avanafil is superior to sildenafil in having a faster onset of action with a shorter time to peak response (20-40 min after avanafil dosing vs >60 min after sildenafil dosing) and improving erectile function within 15 min, which reduces the inconvenience regarding dosing time.^9,10,15^ In this case series, the patients reported an onset time of 15 min upon taking the medication, which allowed them to have better overall sexual experiences. Therefore, avanafil can be taken 15-30 min before sexual intercourse instead of 1-2 h before, similar to other PDE5is.

Given the data on the safety and efficacy of avanafil, healthcare professionals should consider this medication as a potential treatment option for patients with ED, tailoring the dosage based on individual needs and co-morbidities. The choice between 100 mg and 200 mg avanafil should be based on patient characteristics and response to initial therapy. By addressing ED, avanafil contributes to enhancing patients’ quality of life, psychosocial well-being and overall satisfaction.^4^ Improved sexual function positively impacts self-esteem, confidence and intimacy, which are essential aspects of holistic health.^20^

Despite the positive outcomes observed in the 200 mg avanafil treatment group, there was no significant difference noted in the overall avanafil treatment group (100 mg + 200 mg). Notably, the SD of the IIEF-5 scores was substantially high in this case series, as standardisation of the baseline IIEF-5 scores was not possible due to the limited number of study samples. Hence, the limited sample size indicates the need for future studies with larger cohorts to validate the findings and explore potential subgroup differences. Moreover, while the short-term results are promising, longterm follow-up is crucial to assess the sustained efficacy and safety of avanafil.

## Conclusion

Avanafil emerges as a valuable treatment option for patients with ED, particularly those with comorbidities. Further research should focus on expanding the evidence base, considering diverse populations and addressing long-term outcomes.
